# The underlying reasons for very high levels of bed net use, and higher malaria infection prevalence among bed net users than non-users in the Tanzanian city of Dar es Salaam: a qualitative study

**DOI:** 10.1186/s12936-017-2067-6

**Published:** 2017-10-23

**Authors:** Daniel Msellemu, Aloysia Shemdoe, Christina Makungu, Yeromini Mlacha, Khadija Kannady, Stefan Dongus, Gerry F. Killeen, Angel Dillip

**Affiliations:** 1 Environmental Health and Ecological Sciences Thematic Group, Ifakara Health Institute, Kiko Avenue, Mikocheni, PO Box 78373, Dar es Salaam, United Republic of Tanzania; 2 Department of Epidemiology and Public Health, Swiss Tropical and Public Health Institute, Socinstrasse 57, 4051 Basel, Switzerland; 3 Faculty of Natural Science, University of Basel, Petersplatz 1, Postfach 4003, Basel, Switzerland; 4 Dar es Salaam City Council Ministry of Regional Administrations and Local Government, Dar es Salaam, United Republic of Tanzania; 5 Department of Vector Biology, Liverpool School of Tropical Medicine, Pembroke Place, Liverpool, L3 5QA UK

## Abstract

**Background:**

Bed nets reduce malaria-related illness and deaths, by forming a protective barrier around people sleeping under them. When impregnated with long-lasting insecticide formulations they also repel or kill mosquitoes attempting to feed upon sleeping humans, and can even suppress entire populations of malaria vectors that feed predominantly upon humans. Nevertheless, an epidemiological study in 2012 demonstrated higher malaria prevalence among bed net users than non-users in urban Dar es Salaam, Tanzania.

**Methods:**

Focus group discussions were conducted with women from four selected wards of Dar es Salaam city, focusing on four major themes relating to bed net use behaviours: (1) reasons for bed net use, (2) reasons for not using bed nets, (3) stimuli or reminders for people to use a bed net (4) perceived reasons for catching malaria while using a bed net. An analytical method by framework grouping of relevant themes was used address key issues of relevance to the study objectives. Codes were reviewed and grouped into categories and themes.

**Results:**

All groups said the main reason for bed net use was protection against malaria. Houses with well-screened windows, with doors that shut properly, and that use insecticidal sprays against mosquitoes, were said not to use bed nets, while frequent attacks from malaria was the main stimulus for people to use bed nets. Various reasons were mentioned as potential reasons that compromise bed net efficacy, the most common of which were: (1) bed net sharing by two or more people, especially if one occupant tends to come to bed late at night, and does not tuck in the net 71%; (2) one person shares the bed but does not use the net, moving it away from the side on which s/he sleeps 68%; (3) ineffective usage habits, called *ulalavi*, in which a sprawling sleeper either touches the net while sleeping up against it or leaves a limb hanging outside of it 68%. Less common reasons mentioned included: (1) Small bed nets which become un-tucked at night (31%); (2) Bed nets with holes large enough to allow mosquitoes to pass (28%); and (3) Going to bed late after already being bitten outdoors (24%).

**Conclusions:**

Behaviours associated with bed net use like; bed sharing, bed net non compliant-bedfellow, sleeping pattern like *ulalavi* and some physical bed net attributes compromise its effectiveness and supposedly increase of malaria infection to bed net users. While some well-screened houses looked to instigate low malaria prevalence to non-bed net users.

## Background

All over the tropics, local understandings of malaria, as a disease and an infection, are varied and often far more socially complex in human terms than simple consideration of the *Plasmodium* parasite’s life cycle would suggest [1]. One of the most successful interventions against malaria has been the introduction of bed nets. Even before bed nets were treated with insecticide they reduced malaria infection firstly as a physical barrier, and had mass effects on mosquito population density, survival, infection prevalence and vectorial capacity [2–4]. The introduction of long-lasting insecticidal nets (LLINs) and policy that focus on community protection from malaria vectors rather than personal protection of vulnerable groups like pregnant women and children has helped to reduce malaria cases [5–7]. The scale up of such interventions has reduced morbidity and mortality from malaria for the last 15 years [8].

Tanzania had a well-established bed net distribution campaigns. which began in 2004 with the Tanzania National Voucher Scheme (TNVS) [9, 10], followed by two rounds of mass distribution campaigns between 2009 and 2011 and achieved universal coverage of 68, 73 and 76% of household-members, under-five and pregnant mothers respectively by 2012 [11]. Furthermore, bed net use in Dar es Salaam, the largest city in Tanzania, considerably exceeded national average, with > 90% usage observed by two independent surveys [9].

Because of the physical barrier property and impregnated insecticide, which also repels mosquitoes, bed net users are usually observed to have lower malaria prevalence than non-users [12]. Bed net users are individuals who had verbally reported to use bed net in the previous night during the 2010–2012 cross sectional survey and the bed nets were visually confirmed by a study surveyor to be present and hanged at the bed [13]. Non bed net users are individuals who reported not to have been using bed nets during the said cross sectional survey. An absence of a bed net was also confirmed. The cross-sectional survey of malaria infection prevalence in urban Dar es Salaam between 2010 and 2012 surprisingly found that, at individual level, bed net users had higher malaria infection prevalence than non-users [13]. Furthermore, similarly surprising and counter-intuitive observations of a positive association between bed net use and malaria infection status have also been reported in other studies [14] on malaria infection prevalence and where massive bed net campaigns had no effect [15, 16]. The qualitative study was, therefore, conducted to explore underlying reasons for individual bed net use and non-use among the residents of Dar es Salaam, and elucidate the causes of the observation that malaria prevalence appears to be higher among bed net users.

## Methods

### Study design

This was a qualitative study, rooted in the principles of grounded theory. The study followed the principles of consolidated criteria for reporting qualitative studies (COREQ) [17, 18]. Focus group discussions (FGDs) were used to collect information from study participants, with an inductive approach that allowed participants to share their perceptions on issues related to use and non-use of bed nets, after which participants were probed for other necessary information, which were predefined in the interview guide.

### Setting

**Fig. 1:**
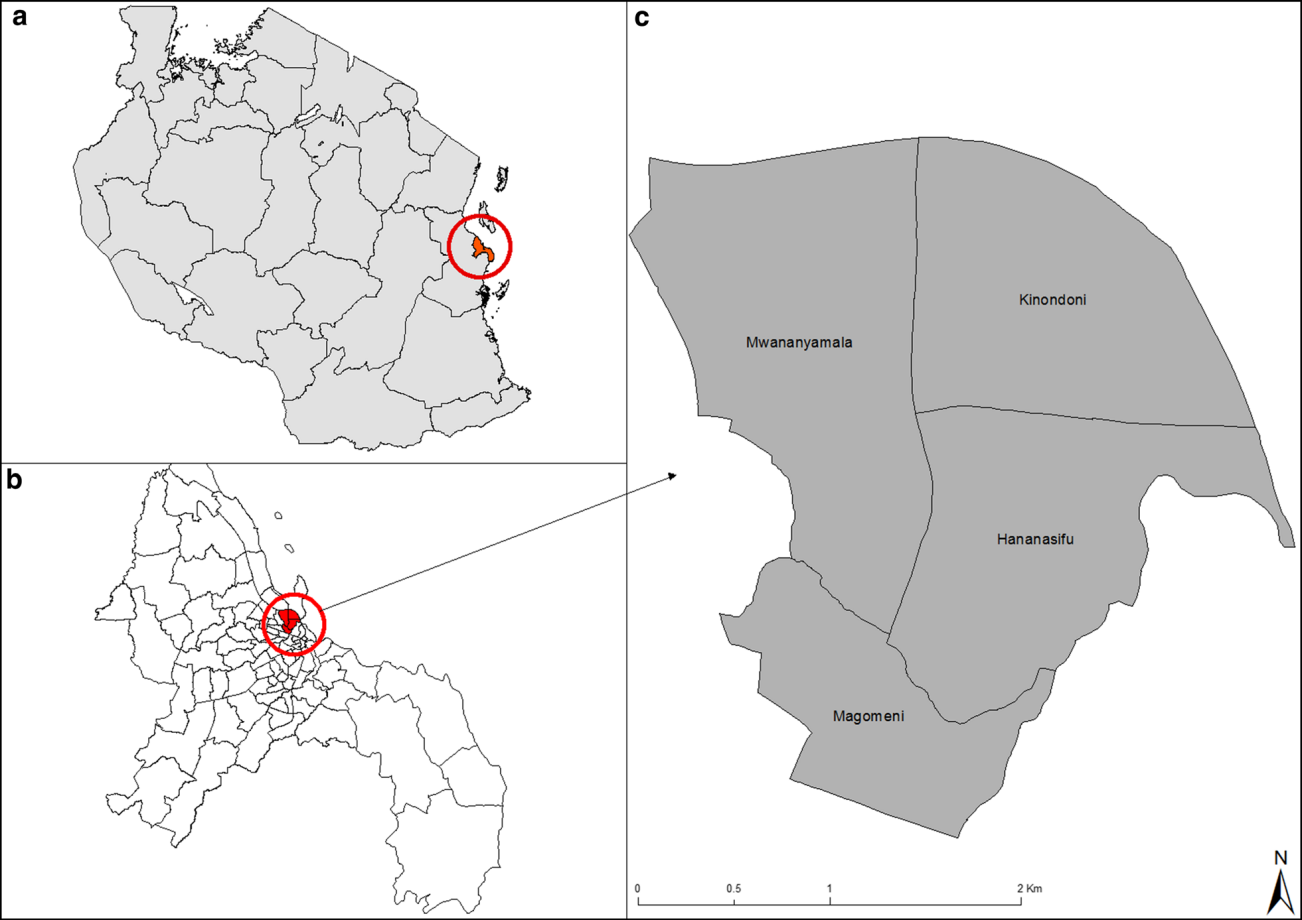
Map of the study sites. **a** Map of Tanzania showing Dar es Salaam city; **b** map of Dar es Salaam city showing administrative wards of the city; **c** the four wards the focus group discussions were conducted in

The study was conducted in four wards of the Dar es Salaam city which were sampled by the second round of cross-sectional parasitological surveys as described previously [13], between September 2010 and August 2012. Specifically, the FGDs were conducted in housing clusters, referred to as Ten-Cell Units or *mashina*, previously sampled [13] in the wards of Mwananyamala, Hananasif, Kinondoni and Mkwajuni, between August and September 2012 (Fig. 1).

These wards were purposively chosen due to their socio-cultural homogeneity [18–20], because they include some of the oldest settlements in Dar es Salaam [21]. The wards have ethnically diverse resident populations, originating from most of the tribes and regions of the country, that have been homogenized into a single community through a long-term co-residence and intermarriages in these long-settled urban contexts. These four wards also have similar environmental characteristics and distributions of socio-economic activities. The common household income-generating activities in the study wards include street-vending [22, 23] (selling of home-made products, such as bread, cakes, doughnuts, boiled and fried cassava and potatoes), small-scale businesses, retail shops and other informal sector trade and services.

### Data collection procedures

Prior to data collection, a pilot FGD exercise was conducted, following which the interview guides were revised and later used for the actual formal data collection. An experienced moderator who is a senior social scientist facilitated the FGDs [18] in Kiswahili, assisted by two assistant research scientists who took notes and recorded the discussion. The discussions were based around key themes [18] relating to factors influencing bed net use and non-use. Discussions started with general questions related to factors driving such high levels of routine bed net use, and then focused on particular questions regarding general experience about bed nets, reasons for not using bed nets, and factors that stimulate or reminds people to use bed nets. Data were collected using a theoretical saturation approach [24–26], whereby data were collected until no additional information was generated.

### Participant selection

The study included four groups of participants who were chosen based on their *Plasmodium falciparum* infection diagnostic status, bed net use habits and the presence or absence of complete window screening in their houses, as recorded in previous parasitological surveys [13]. These participant groups were composed with reference to individuals malaria diagnosis during the 2012 epidemiological survey [13] as follows: Group one in Kinondoni Ward were all diagnostically confirmed to have been malaria-infected, reported having used a bed net the previous night before the survey, and lived in houses with complete window screening. Group two in Mkwajuni Ward were all malaria-infected and lived in houses with complete window screening but reported not having used a bed net the previous night before the survey. Group three in Hananasif Ward all slept in houses with complete window screening and had used a bed net the previous night before the survey but had no detectable malaria infection. Group four in Mwananyamala Ward had all slept in a house with complete window screening but had no detectable malaria infection and had not used a bed net the previous night before the survey. All FGD participants from the four groups were diagnostically confirmed to be either malaria positive or negative during the cross sectional survey [13]. Purposive sampling was used to ensure that residents of the exact housing clusters sampled in previous parasitological surveys who matched the inclusion criteria were traced and recruited for the FGDs, specifically consenting individuals who previously answered the full questionnaire and been tested for malaria infection [13] (Table 1).

**Table 1:** Composition of focus groups

No	Type of group	Ward	Discussion held	Age average	Gender	Participants
1	Malaria positive, bed net absent, window screen present	Mwananyamala	1	[27–59]	F	12
2	Malaria negative, bed net absent, window screen present	Hananasif	1	[24–56]	F	10
3	Malaria positive, bed net present, window screen present	Kinondoni	1	[31–78]	F	12
4	Malaria negative, bed net present, window screen present	Mkwajuni	1	[22–48]	F	11

Inclusion criteria for participation in the study were; being an adult woman of 18 years or above, and either a head of a household or a wife of the head of a household or the oldest woman in a household. Study participants also had to have answered the full questionnaire and been tested for malaria infection during cross-sectional parasitological survey [13]. These inclusion criteria were developed based on the finding that > 60% of the respondents in the preceding cross-sectional parasitological survey were women [13], who are usually more likely to be at home during daytime household surveys, and have the greatest opportunity to directly observe the behaviour and activities of most household members through their routine household management roles. The number of participants per each FGD ranged from 10 to 12 women. The discussions were carried out at the respective local government ward offices or other venues in their immediate surroundings (Table 1).

### Data analysis

Audio recordings were transcribed and translated into English by a bilingual research scientist speaking both Kiswahili and English, whose first language was the former. Two researchers coded the data, checked for consistency, and identified units of information that covered broad categories. Analysis was undertaken using a framework grouping of relevant themes, to address key issues of relevance to the study objectives [27]. Lists of codes were reviewed and grouped into categories and themes for analysis. Representative quotes chosen were the ones that mostly represent study findings and better reflects study objectives and research findings [28, 29].

## Results

### Reasons for bed net use

Regarding perceived drivers for bed net use in the participants’ households and community, unanimously across all four groups, all participants who responded to this question on bed net said bed nets are specifically for protection against malaria:

“*We use bed nets to protect ourselves against malaria*” (Mkwajuni, participant 1)

Participants went further to explain how bed nets also protect against flies and other nuisance insects, as well as cold weather as narrated below:

“*We also use bed nets to protect ourselves from flying insects like houseflies, and also against cold weather*” (Ukwamani, participant 2)

Bed nets thus also protected users against non-biting pests like cockroaches and other crawling insects when asleep. Other reasons cited for bed net use included prevention of dust getting onto bed sheet and coverings and to prevent people from falling out of bed while sleeping, especially when they are dreaming.

### Reasons for not using a bed net

The next theme discussed was perceived reasons for not using a bed net. Participants gave their own experiences of reasons for not using a bed net and those of other household members, but also referred to the community they live in. By far the most common and immediate spontaneous answer to the question “Why do you think some people do not use a bed net?” was that people cannot afford to buy bed nets. This was the same across all four groups;

“*Some cannot afford to buy a net. They cannot even feed their household well, let alone buying a bed net*” (Ukwamani, participant 9)

“*Some cannot afford to buy a bed net. They find them expensive*” (Hananasif, participant 7)

When the participants were asked about the bed nets that were provided freely to all residents by the mass distribution campaigns carried out over the previous 2 years [30], affordability was no longer considered an issue, but rather other social and behavioural factors emerged as contributors to non-use of bed nets. The most common response that the majority of participants agreed as a reason for not using bed nets when access was not an issue was “presence of window screening” and “use of mosquito sprays”. However, the participants unanimously agreed that houses with window screening protect people when they are inside houses. Furthermore, participants explained how some individuals live without using a bed net but do not experience malaria. In fact, they emphasized the protective value of well-screened houses, closing doors properly in the evenings and alternative personal protection measures like mosquito repellents. These were perceived to be main reasons why some people who do not use bed net still do not get malaria.

“*People have well*-*made houses. Their houses have multiple doors before you get to the bedrooms. Their front and back doors have mosquito mesh and their roofs have well*-*sealed ceilings. On top of that, they also use mosquito repellents. Such households do not use bed nets. Being bitten by a mosquito in such houses is very unusual. But many people cannot afford such houses*” (Hananasif, participant 3)

“*Some use sprays and believe that it’s enough, so they do not need a bed net*” (Hananasif, participant 9)

“*Some shut their doors properly so that mosquitoes cannot get into the house. These kinds of people may sleep without bed nets and will not be bothered by mosquitoes to a great extent*” (Mwananyamala, participant 2)

“*But there are fewer homes of this type, the houses that can be closed properly to prevent mosquitoes from getting in*” (Mkwajuni, participant 10)

It was again reported that, some families actually buy bed nets, but do not use them even after spending their own money on the purchase. These bed-nets were said to be used only for a visitor to sleep under while staying with the household. Other families were said to buy nets to be used by their children.

“*Yes, people buy bed nets for their children and family’s visitors to use, but not for other household adults use*” (Mkwajuni, participant 1).

Some people may despair on their effort against malaria; this was noted in the Mwananyamala group when a participant said:

“*Others may not use bed nets because of frequent attacks of malaria. Some had constantly used bed nets but they constantly get malaria. This leads to despair because they feel that the nets do not work. They feel that bed net use has not prevented them from getting malaria and so they become negligent. These kinds of people are those who are also diagnosed with malaria every time they get to the hospital*” (Mwananyamala, participant 3)

However this opinion was strongly challenged by other group members who said, such experience would motivate people to use bed nets far more than to not use them, because people can get malaria elsewhere and not all malaria is related to non-use of bed nets.

### Stimuli and reminders to use bed nets

Previous exposure to malaria was consistently perceived as the major reason for household members to use bed nets. All participants from the four discussion groups said the experience of being sick, as well as the costs for malaria treatment and other expenses associated with caring for a sick person, prompts them to use bed nets.

“*It is malaria that reminds us to use bed nets. Those who have been suffering from malaria remember the most to use bed nets. Just like someone under medication remembers to take his medicine*” (Ukwamani, participant 6)

“*The family that is frequently attacked by malaria tends also to use bed nets frequently. Treatment is expensive, so the family tries to protect itself against malaria. Therefore, repeated malaria attacks compel the family to buy bed nets. Treatment is expensive and it is more expensive if many family members are involved. In order to avoid this, we buy bed*-*nets*” (Mwananyamala, participant 3)

“*It is malaria. When you get malaria repeatedly, you will end up buying a net. You just give up and try to avoid death. Many like to buy bed nets but they are expensive. The freely given nets are not easy to get and some are small, and do not fit in most beds*” (Mkwajuni, participant 1)

“*There are people out there who use bed nets regardless of malaria infection. However, generally people who have experienced malaria tend to use bed nets more*” (Hananasif, participant 11)

Those who have used bed nets for many years said that the practise of bed net use has become a habit to them and they cannot sleep without. To them, the use of bed nets is not influenced by wet or dry season, they constantly use bed nets. Use of a bed net is also regarded as being associated with personal hygiene, concern with one’s health, and how well a person has been raised.

“*People who take good care of their health usually use bed nets. Some are raised that way and they are likely to always use a bed net, without needing to be reminded*” (Mwananyamala, participant 6)

Others mentioned health education campaigns as important reminders to use bed nets.

“*Health education on malaria also reminds us to use bed nets. Sometimes one can forget, and does not give net use a high priority, but when you hear from the campaigns about the importance of bed net use and the consequences of not using one, you immediately buy or start using a bed net*” (Ukwamani, participant 8)

For some participants, however, bed net use was considered seasonal. These participants reported to remember using bed nets during the rainy season, because there is increased nuisance from biting mosquitoes and this is a factor that reminds them to use bed nets.

### Perceived reasons for catching malaria despite using a bed net

Next, perceived reasons for acquiring malaria despite using bed nets were discussed. The most commonly cited reasons for malaria episodes among bed-net users were various consequences of sharing a bed net (Table 2) the details of which are explained as follows.

“*People who share bed nets are more likely to get malaria than those who don’t. For example a man and his wife; the man may tuck*-*in the net but the wife may un*-*tuck it simply because she does not like it. One part of the net will be hanging loose. As they keep sleeping later at night the whole bed net is left loose*” (Mwananyamala, participant 8)

“*Some have bed nets and use them, but if you share a bed with someone and s/he comes late to sleep, and gets into the bed carelessly and does not tuck*-*in the net correctly, then mosquitoes get inside*” (Mkwajuni, participant 7)

**Table 2:** Common reasons said to compromise bednet efficient among bed net users

Ward	Bed net shared, one occupant comes late to sleep, and does not tuck in the net		One person shares the bed but does not use the net, pushing it away to other side		Ineffective bed net use habits, *ulalavi*, sleeper touches the net or leaves a limb hanging outside of it		Small bed nets which become un-tucked at night		Bed nets with holes large enough to allow mosquitoes to pass		Going to bed late after already being bitten outdoors		N
	n	(%)	n	(%)	n	(%)	n	(%)	n	(%)	n	(%)	
Mwananyamala	10	83	9	75	7	58	4	33	3	25	3	25	12
Hananasif	7	70	6	60	8	80	2	20	3	30	4	40	10
Kinondoni	9	75	8	67	10	83	5	42	5	42	2	17	10
Mkwajuni	6	54	8	73	6	55	3	27	2	18	2	18	12
Overall (%)		71		68		68		31		28		24	

Horizotal parcenteges are based from the responce within a focus discussion group. (Overall(%)) vertical percentage are collective respoce across all four foucus discussion groups

Another mentioned reason was the fact that the bed nets are too small. Such expressions of dissatisfaction were particularly directed to the free nets provided by the National Malaria Control Programme through two sequential rounds of mass distribution [30, 31]. Interestingly, these freely distributed nets were often referred to in Kiswahili as *Neti za Bush*, meaning bed nets from President Bush in reference to funding support for their procurement by the United States President’s Malaria Initiative. Participants in all four groups complained that these nets were small compared to their bed size, and they had mesh size holes larger enough to allow mosquitoes to pass through. They highlighted these factors as reasons why some people did not use the freely distributed nets. They also mentioned that those who do use them often wake up in the morning to find the bed net no longer tucked in.

“*Some of the bed nets have larger holes and allow mosquitoes to go through. One day we observed this: A mosquito tried to get into the net and eventually succeeded*” (Ukwamani, participant 5).

Interestingly, un-tucking bed nets after waking up in the middle of the night and early morning was also perceived as another reasons for catching malaria despite using a bed net. It was said that mosquitoes that managed to get inside the bed net at night remain trapped in the net until the net is un-tucked so that they can escape. Some net users were reported not to un-tuck their bed nets in the morning and leave them hanging down over the bed for whole day. The trapped mosquitoes remain inside, readily waiting to bite when someone returning to sleep in the evening. The participants reasoned that if the trapped mosquitoes have malaria parasites, they could pass the infection to a person sleeping inside the net the next night.

“*Also some tuck in their bed nets but do not take it out [un*-*tuck it] in the morning. So if there are mosquitoes trapped inside it, they remain and will probably bite the owner on the second night when s/he comes to sleep*” (Hananasif, participant 5)

‘Dirty bed nets’ were another perceived reason for catching malaria among net users. Several participants suggested that some bed nets were hung but not used because they were dirty. It is said that people do not like to use bed nets that have been hung for a long time without being washed. Some respondents reported that when the cross-sectional parasitological questionnaire was administered [13], people with such habits sometimes claimed to have slept under bed nets and showed the survey team the net, but in reality the net was never used.

“*Because of cleanliness, some husbands do not like dirty bed nets. They want nets to be washed every few days. So if he gets home and finds that the bed net has not been washed, he will put it away. This may result the family or couples sleeping without a bed net*” (Mkwajuni, participant 9)

Travelling and staying away from home was also said to cause malaria amongst bed net users. While travel away from home has, counter-intuitively, been shown to be associated with higher malaria infection risk in two independent cross-sectional parasitological studies [13, 32], participants in this qualitative study consistently emphasized that they come back with malaria when they travel far away from home.

“*Sometimes we travel and some homes that host us may have no bed nets, so when you stay for some days in these homes you are likely to get malaria*” (Hananasif, participant 1)

Consistent with epidemiological evidence from the preceding cross-sectional surveys [13], participants from all the groups shared the perception that people get malaria by being bitten by mosquitoes before they go to bed and, more often, before they even get to their homes. Most participants confirmed staying outdoors for some hours after dark before going indoors for the evening, and some up to 1:00 a.m. for various reasons [13]. Common reasons were cooking outdoors, watching football on television and nearby video-cabins within their neighbourhood. Video cabins are big wooden constructed kiosks with a hall large enough to accommodate between 20 and 40 people watching live football matches or cinema for a price of only TSh 500 (US$ 0.30). They have a roof but no ceiling and the eaves are usually not screened.

“*For example, I use a bed net and I still get malaria. But the nets do not cause malaria, it may be because I stayed outside and got bitten or I was bitten when I was indoors before going to bed. I may still get bitten while indoors unless I go to bed and draw a net*” (Mwananyamala, participant 9)

Unusual or ineffective use of bed nets was also reported as a reason for catching malaria despite bed net use. It was noted that some people were bitten while they slept leaning up against the bed net. Also, others leave parts of their body outside the bed net, such as the hand, foot or face. Intentionally putting the face outside the bed net was commonly cited as a deliberate attempt to get a breath of fresh air from outside the net. Participants reported that others are not protected by bed nets because they either simply forgot to use or because they tucked in the net improperly, which allowed mosquitoes to get inside the bed net.

“*Most people use nets, but they use them differently to the way they are supposed to. For example, my mother will fix the net on the bed but she will leave her face outside of the bed net. She has difficulty in breathing when she sleeps fully under the bed net. She gets bitten on the face, and in the morning we find some mosquitoes inside the net too*” (Ukwamani, participant 8)

## Discussion

The primary goal of this study was to explain why people who use bed nets in Dar es Salaam have higher malaria infection prevalence than people who do not [13]. While bed nets are known to be highly protective against malaria [12, 33, 34], and obviously do not cause malaria among users, the counter-intuitive epidemiological observation that non-users have lower malaria infection prevalence than users in this setting requires some explanation.

Most of the participants from four groups exhibited knowledge, attitudes and practices with respect to bed net use. Indeed, “protection against malaria” was the most commonly mentioned reason for using a bed net. It seems that households primarily obtain and use bed nets to reduce malaria morbidity and associated treatment costs among household members. Participants also mentioned additional reasons for bed net use, which include protection from a wider diversity of insects like cockroaches and other household pests, falling out of bed while asleep, dust and cold weather. Nevertheless, the previous, sometimes repeated experience of malaria illness was considered to be a motive for households to use bed nets more frequently. These documented perceptions and perspectives of the participants illustrate how prevalence of malaria among bed net users may be higher than among non-users, because routine bed net use is viewed favourably based on their personal past malaria experiences. The epidemiological observation of higher malaria prevalence among bed net users may therefore be rationalized in terms of reverse causality: malaria causes higher bed net use probability. Attempts to measure the negative association between net use and malaria infection may be confounded and even reversed by the positive association arising from illness-motivated bed net use.

Consistent with previous studies [35–37] and the perceived role of bed nets as a means to protect against mosquitoes and malaria, use of bed nets was also reported to be higher during the rainy season. The findings that bed nets are not used by all household members, but instead reserved for children and visitors is also consistent with previous studies describing sleeping arrangements and bed net allocation priorities within families [38, 39].

The perception that sharing bed nets limits their protective effect is consistent with some epidemiological studies demonstrating that children who share beds nets experienced more hospital visits for malaria treatment than those who did not [40]. Some of the reasons that may explain why bed net sharing reduces protective efficacy of bed nets are that humans synergistically attract more mosquitoes per person when they aggregate into groups. Although the probability of one being bitten may be reduced due to number of people, the probability of a mosquito getting a successful bite increases [41]. If one of the persons sharing a bednet has *ulalavi*, the tendency to sprawl while sleeping with legs and arms scattered so that they touch the net or hang out of it is considered to increase biting exposure. Risk of exposure in contact with or outside of the net is also exacerbated when two or more people share a bed, so they are unlikely to cluster at the middle of it for reasons of personal comfort. Instead they will tend to sleep to one side, much nearer to the bednet than they would if sleeping alone in the centre of the bed [41, 42].

Catching malaria despite using bednets was also perceived to be associated with spending evenings outdoors [43, 44], often for social activities like resting outdoors, communal-television watching, and public drinking venues simply because these are common activities people do when they are outside in areas such as Dar es Salaam.

Participants did not tell of any particular means of self-protection against mosquito bites while outdoors when resting, watching television or at outdoor bars. This is suggesting substantive gaps in the availability and awareness of vector control measures for preventing outdoor malaria transmission which clearly does occur in this setting [13]. Several new or under-exploited intervention options for addressing residual malaria transmission, including methods for personal protection against exposure, have become available [45] and the World Health Organization now encourages their evaluation [34].

Complex reasons were mentioned with regard to non-use of bed nets, and while affordability was mentioned, social and behavioural reasons were more important. Participants expressed their concern that not using a bed net will result in getting malaria, unless one can afford and effectively use mosquito repellents or insecticidal sprays, or stay in a house with screened windows and doors that shut properly.

Participants argued that although good houses are a better means for malaria prevention than bed nets or sprays, few people live in such houses. Interestingly, this is inconsistent with the results of the much larger scale cross-sectional study these study locations and focus groups were selected from, which showed that the majority of residents lived in houses with complete window screening, albeit with some holes in most cases [13]. This suggests that either the focus groups selected came from parts of the city with unusually low coverage of window screening, or that perceived protection against mosquitoes generally differs from *de facto* protection against *Anopheles* malaria vectors specifically. Given that the vast majority of human-biting mosquitoes that motivate self-protection against mosquitoes in Dar es Salaam [46] are *Culex quinquefasciatus* that do not transmit malaria, and the majority of complete window screens provided protection against malaria and therefore *Anopheles* despite having holes, the latter explanation seems more probable but remains to be confirmed and explored. Further mixed-method studies comparing community perceptions with entomological surveys of their actual exposure to biting mosquitoes of all types in different times and places may provide valuable insights into motivators for personal or household protection. Such mixed-method studies may also be useful to help understand the perceived value placed on programmatic implementation of different vector control methods, all of which selectively target specific mosquito taxa to some extent. This may be especially important in urban settings like Dar es Salaam, where malaria-transmitting *Anopheles* comprise only a small minority of the human-biting mosquito population [46].

While this study does provide several useful insights that can be used to consolidate and improve upon the impact of bed nets in this setting, the general relevance of these observations beyond Tanzania and even Dar es Salaam is unclear. Furthermore, this is an observational rather than experimental study, and so can only be described as evidence of *plausibility*, rather than *probability* [47], that malaria prevalence is higher amongst bed net users because experience of malaria motivates uptake. Additional issues raised by this study, but which will require further study before conclusive policy recommendations could be formulated include; (1) the discrepancy between perceived coverage and impact of mosquito-proofed housing versus the high levels already documented epidemiologically, (2) product selection and delivery options for improving bed net availability, size, durability and physical protection, (3) behaviour change communication strategies for improving bed net use practices, and (4) intervention options for preventing outdoor malaria transmission.

## Conclusions

Despite these study limitations, this selective, small-scale application of qualitative social science methods to follow up on a large-scale, comprehensive cross-sectional quantitative study of malaria risk [13], has enabled rationalization of the counter-intuitive observation that higher malaria prevalence occurs amongst residents who use bed nets than those who do not. The sequential combination of these two complementary approaches has allowed us to explain the somewhat alarming positive association between malaria infection and bed net use, which was initially revealed by the quantitative study but could not be clearly understood without this complementary qualitative study. It is now reassuring to conclude that malaria prevalence is higher amongst bed net users than non-users in Dar es Salaam, precisely *because* they perceive them to be highly protective against malaria.

Furthermore, this qualitative study has allowed identification of several additional important issues for further research, and even for direct translation into policy and practice. Several sub-optimal usage practices for bed nets compromise their effectiveness as personal protection measures, and most of these could be addressed through behaviour change communication programmes. However, to enable improved usage practices, bed net programmes in Tanzania will need to increase distribution rates to minimize the necessity for bed net sharing. They should also prioritize procurement of larger nets, which are durable and easy to fit onto beds, with holes small enough to prevent mosquito entry. The protective effect of bed nets is also clearly limited to sleeping hours and indoor environments, so evaluations of additional measures to protect against outdoor mosquito bites need to be evaluated, inclusive of user perceptions and uptake.

## Abbreviations

FGDs: focus group discussions
IHI: Ifakara Health Institute
IRB: Internal Review Board
LLINs: long lasting insecticidal nets
NIMR: National Institute of Medical Researches
TNVS: Tanzania National Voucher Scheme
TShs: Tanzania Shillings–National Currency
